# Supplementary material for: Brain age prediction using deep learning uncovers associated sequence variants
Source: Nat Commun. 2019 Nov 27;10:5409. doi: 10.1038/s41467-019-13163-9 (PMC6881321; doi:10.1038/s41467-019-13163-9)
Supplement: Supplementary file 1 — Supplementary Information [file 41467_2019_13163_MOESM1_ESM.pdf]

Brain age prediction using deep learning uncovers associated sequence variants

Supplementary Information

Jonsson et al.

Supplementary Figures

Supplementary Figures 1, 2, and 3 show histograms of the distribution of age in the Icelandic dataset, UK Biobank dataset, and IXI dataset.

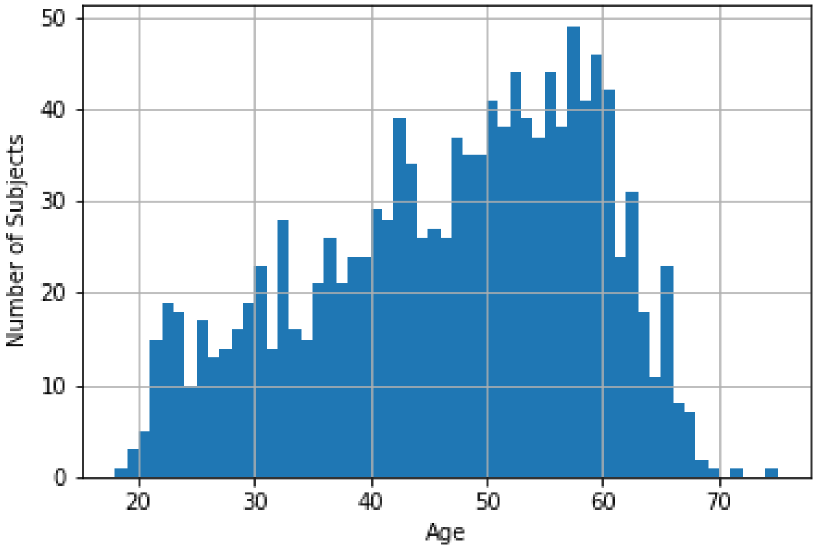

Supplementary Figure 1: A histogram showing the distribution of chronological age in the Icelandic dataset.

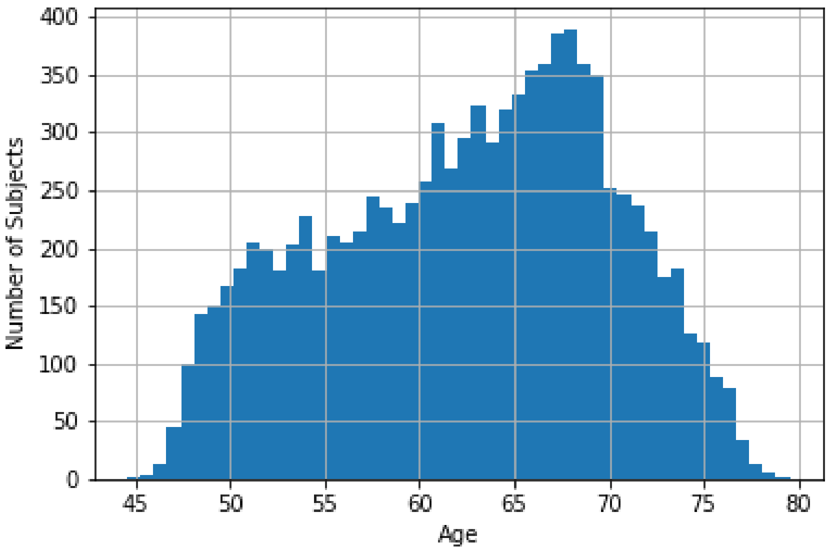

Supplementary Figure 2: A histogram showing the distribution of chronological age in the UK Biobank dataset.

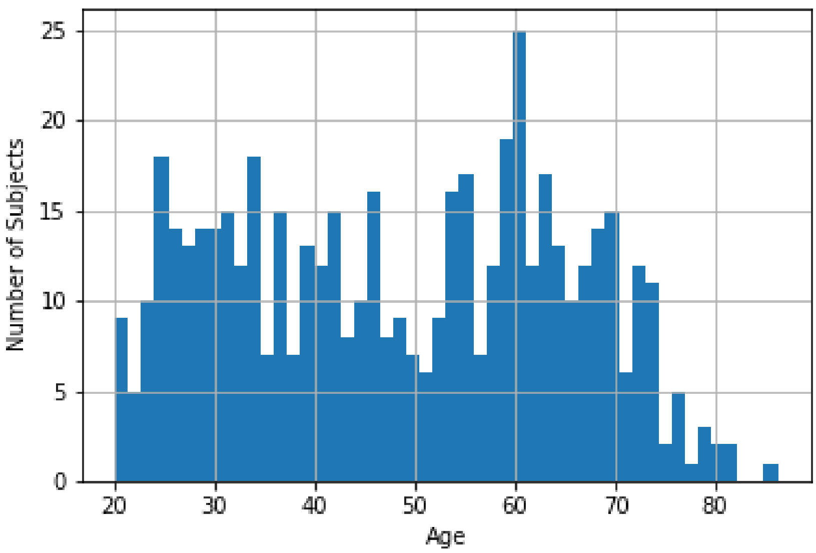

Supplementary Figure 3: A histogram showing the distribution of chronological age in the IXI dataset.

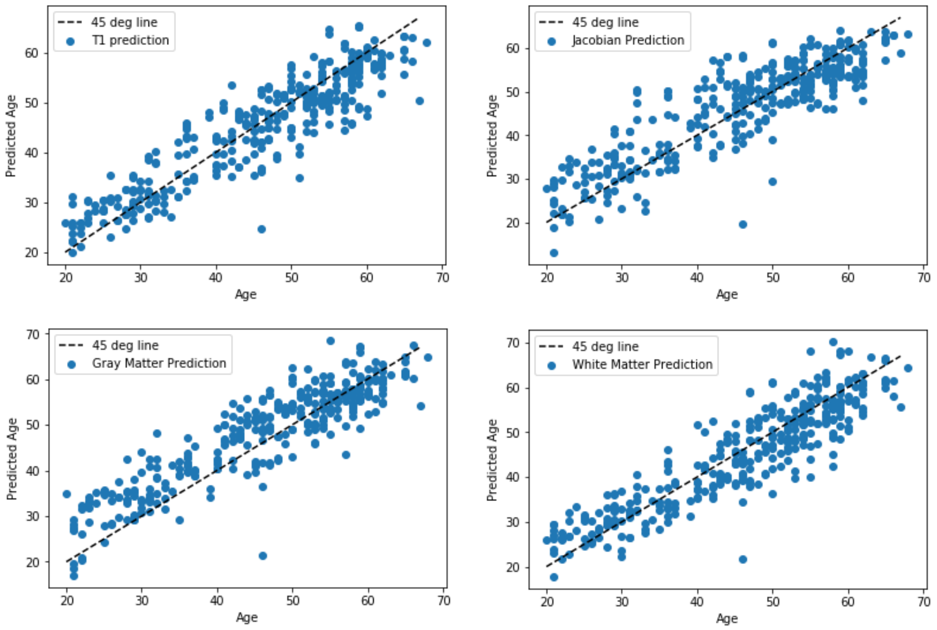

Supplementary Figure 4: Scatter plots showing test set predictions made by CNNs. These plots show the chronological age against the brain age predicted by the CNNs trained on T1-weighted images, Jacobian maps, gray matter segmented images, and white matter segmented images. The top left plot shows the predictions made by the CNN trained on T1-weighted images. The top right plot shows the predictions made by the CNN trained on Jacobian maps. The bottom left plot shows the predictions made by the CNN trained on segmented gray matter images. The bottom right plot shows the predictions of the CNN trained on segmented white matter images.

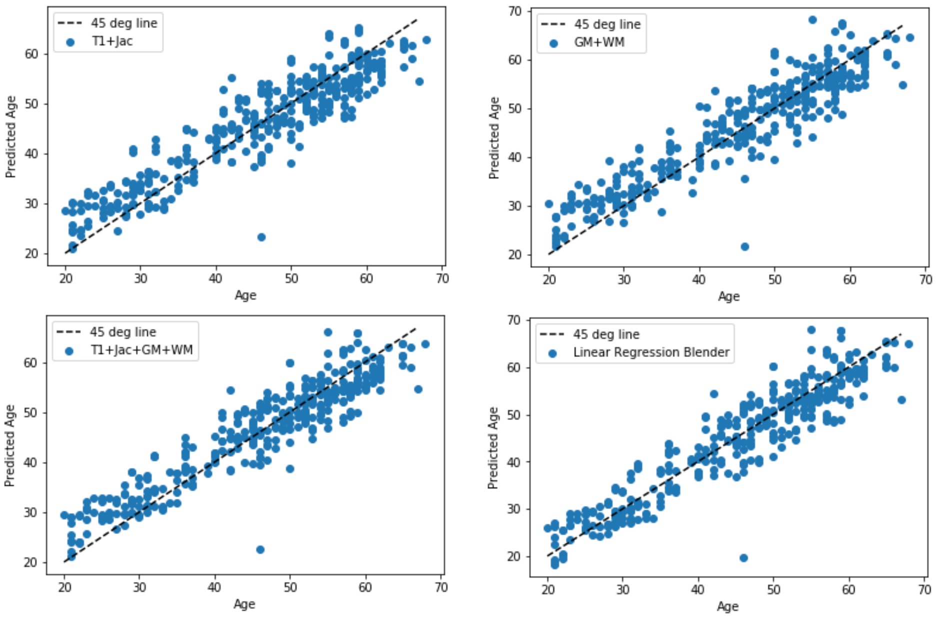

Supplementary Figure 5: Scatter plots showing test set predictions made by combined CNN predictions. These plots show the chronological age against the brain age predicted by a combination of CNNs trained on registered T1-weighted images, Jacobian maps, gray matter segmented images, and white matter segmented images. The top left plot shows majority voting predictions made by the CNN trained on T1-weighted images and Jacobian maps. The top right plot shows the majority voting predictions made by CNNs trained on gray and white matter segmented images. The bottom left plot shows the majority voting predictions made by CNNs trained on T1-weighted images, Jacobian maps, segmented gray and white matter images. The bottom right plot shows the predictions made by the linear regression blender, trained on four predictions from CNNs trained on T1-weighted images, Jacobian maps, and segmented gray and white matter images.

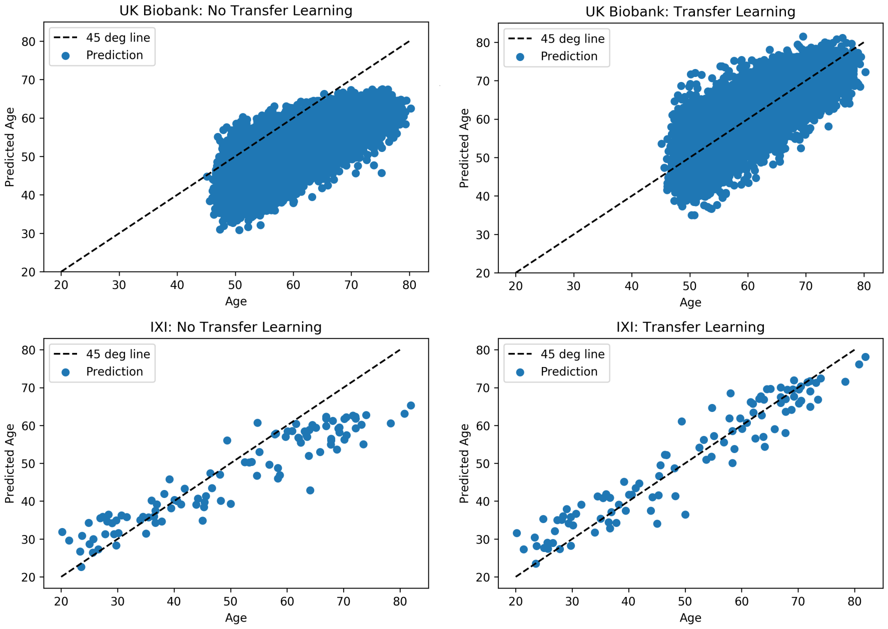

Supplementary Figure 6: Four scatter plots that show the effect of using transfer learning, when predicting brain age of the IXI (validation set) and UK Biobank (test set) datasets, using a CNN trained on images from the Icelandic dataset. These plots show the chronological age against brain age predicted by the CNNs trained on T1-weighted images. The top left plot shows the UK Biobank age predictions without transfer learning. The top right plot shows the UK Biobank brain age predictions with transfer learning. The bottom left plot shows the IXI brain age predictions without transfer learning. The bottom right plot shows the IXI brain age predictions with transfer learning.

Tissue	Samples	NES	p-value	m-value
Pituitary	157	0.167	0.01	0.00
Adipose - Visceral (Omentum)	313	0.131	0.01	0.00
Testis	225	0.124	0.02	0.00
Colon - Sigmoid	203	0.112	0.08	0.00
Pancreas	220	0.0936	0.1	0.00
Prostate	132	0.0686	0.2	0.00
Vagina	106	0.0684	0.4	0.00
Breast - Mammary Tissue	251	0.0434	0.2	0.00
Esophagus - Gastroesophageal Junction	213	0.0420	0.6	0.00
Heart - Atrial Appendage	264	0.0420	0.4	0.00
Heart - Left Ventricle	272	0.0394	0.6	0.00
Skin - Not Sun Exposed (Suprapubic)	335	0.0367	0.4	0.00
Nerve - Tibial	361	0.0303	0.4	0.00
Esophagus - Mucosa	358	0.0239	0.5	0.00
Liver	153	0.0218	0.8	0.00
Adipose - Subcutaneous	385	0.0203	0.6	0.00
Whole Blood	369	0.0190	0.6	0.00
Artery - Coronary	152	0.0170	0.8	0.00
Muscle - Skeletal	491	0.0110	0.8	0.00
Stomach	237	0.0103	0.8	0.00
Colon - Transverse	246	-0.00138	1	0.00
Skin - Sun Exposed (Lower leg)	414	-0.00204	1	0.00
Cells - Transformed fibroblasts	300	-0.00800	0.9	0.00
Artery - Tibial	388	-0.0107	0.8	0.00
Lung	383	-0.0175	0.6	0.00
Thyroid	399	-0.0454	0.3	0.00
Adrenal Gland	175	-0.0476	0.6	0.00800
Minor Salivary Gland	85	-0.0555	0.5	0.00
Esophagus - Muscularis	335	-0.0637	0.3	0.00
Small Intestine - Terminal Ileum	122	-0.0773	0.2	0.00
Artery - Aorta	267	-0.0834	0.06	0.00
Brain - Cerebellar Hemisphere	125	-0.128	0.2	0.192
Brain - Anterior cingulate cortex (BA24)	109	-0.158	0.1	0.140
Brain - Hypothalamus	108	-0.178	0.03	0.472
Brain - Nucleus accumbens (basal ganglia)	130	-0.181	0.1	0.472
Ovary	122	-0.205	0.02	0.628
Brain - Spinal cord (cervical c-1)	83	-0.205	0.02	0.679
Spleen	146	-0.214	0.03	0.464
Brain - Frontal Cortex (BA9)	118	-0.228	0.06	0.564
Uterus	101	-0.246	0.04	0.560
Brain - Cerebellum	154	-0.259	0.01	0.881
Brain - Cortex	136	-0.301	3.2e-3	0.962
Cells - EBV-transformed lymphocytes	117	-0.304	2.7e-3	0.980
Brain - Amygdala	88	-0.317	0.01	0.897
Brain - Hippocampus	111	-0.338	8.0e-5	0.999
Brain - Substantia nigra	80	-0.416	5.8e-5	1.00
Brain - Caudate (basal ganglia)	144	-0.552	1.5e-8	1.00
Brain - Putamen (basal ganglia)	111	-0.565	5.6e-7	1.00

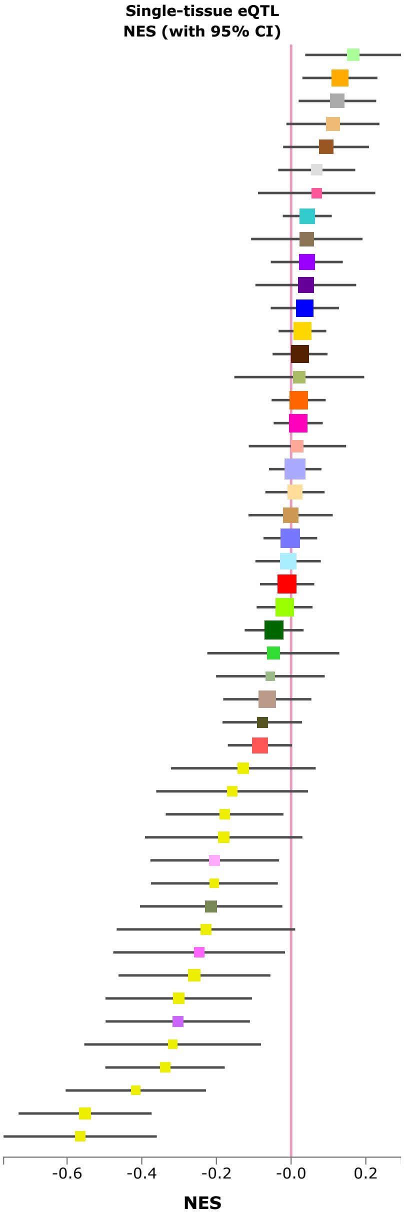

Supplementary Figure 7: Multi-tissue eQTLs comparison for the *RUNX2* gene and rs2790099. Data Source: GTEx Analysis Release V7 (dbGaP Accession phs000424.v7.p2)

The LocusZoom software was used to plot the UK Biobank PAD GWAS results for a 0.2 Mb radius around each of the GWAS significant variants (Supplementary Figures 8 and 9). The LD information was estimated from the European population in the 1000G phase 3 release from Nov 2014. Some of the UK Biobank variants are not present in the 1000G release, these varaints are shown in gray.

As mentioned in the main text, rs2435204 tags an inversion at 17q21.31 (H2), a region of the genome that is known to have high LD [1]. This can be seen in Supplementary Figure 8, which shows that there are many variants in this region that are highly correlated with rs2435204 and reach genome wide significance. From Supplementary Figure 9, we see that rs142628 is located roughly 50 Kb upstream of *KCNK2*.

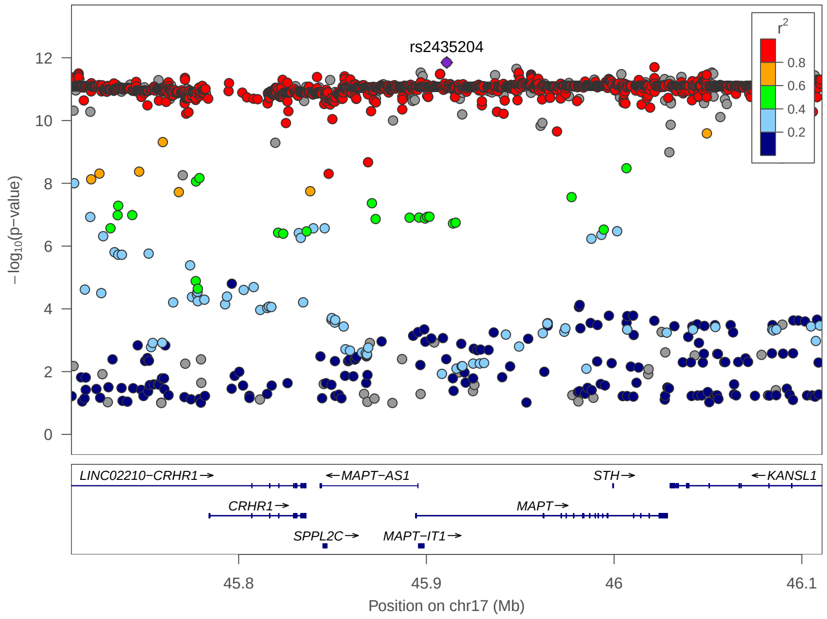

Supplementary Figure 8: LocusZoom plot showing the PAD GWAS results for the neighborhood of rs2435204. SNPs with missing LD information are shown in gray.

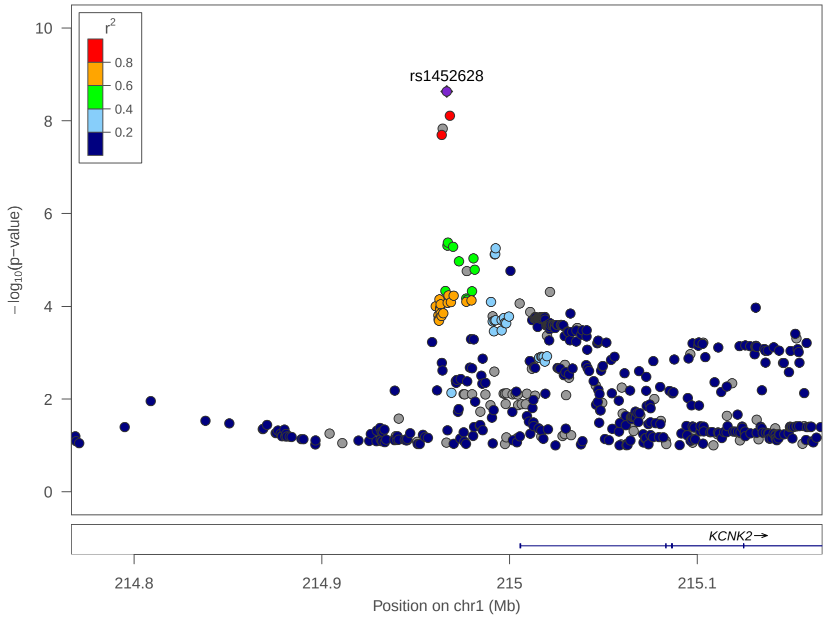

Supplementary Figure 9: LocusZoom plot showing the PAD GWAS results for the neighborhood of rs1452628. SNPs with missing LD information are shown in gray.

Supplementary Tables

Supplementary Table 1 contains results for all of the SBM, VBM, and similarity matrix brain age prediction tests. Some of the tested methods required extensive hyper-parameter tuning, bellow we list the most important parameters of the final models.

When tuning the support vector regression method, we evaluated the performance of two commonly used kernel functions: the linear kernel and the radial basis function (RBF) kernel. The SVR kernel that performed best on the SBM features, i.e. had the lowest test error, was a RBF kernel with $C = 100$ and $\gamma = 10^{-3}$. Similarly, the best performing kernel on VBM features was an RBF kernel with parameters $C = 100$ and $\gamma = 10^{-3}$. The best performing kernel for similarity matrix features was a linear kernel with penalty parameter $C = 10$.

For RVR we tested the same kernel functions as for SVR. Using a linear kernel we achieved the lowest MAE test error for the SBM and similarity matrix features. On the other hand, the RBF kernel had the lowest MAE test error for the VBM features.

For GPR three types of kernels were tested: the dot product kernel, the RBF kernel, and the Matérn kernel. The best performing kernel for the VBM features was a Matérn kernel with length scale $l = 22.0$ and smoothness parameter $\nu = 1.5$. For the SBM features the best performing kerenel was a Matérn kernel with length scale $l = 22.6$ and smoothness parameter $\nu = 1.5$. The best performing GPR kernel for the similarity features was a dot product kernel with $\sigma_0 = 2.0 \cdot 10^{-6}$.

Supplementary Table 1 show that GPR has the lowest test error for VBM input features and SVR the lowest test set error for the similarity matrix features. Additionally, it shows that ridge and elastic net regression have the lowest test set MAE for the SBM features. The ridge regression model that performed best on the SBM features had a regularization parameter equal to $\alpha = 100$. On the other hand, the best performing elastic net model had a $\alpha = 0.1$ and L_1 ratio equal to 0. This means that it is effectively acting as a ridge regression model and explains why ridge and elastic net regression have the same test accuracy when they are trained on the SBM features.

Supplementary Table 1: All SBM, VBM, and similarity matrix results. The cross validation was performed using 10-fold cross validation. Abbreviations: cross validation (CV), Gaussian process regression (GPR), linear regression (LR), ridge regression (RR), elastic net (EN), random forest (RF), relevance vector regression (RVR), similarity matrix (SM), subjects (S), images (I).

Method	Type	CV MAE	CV R^2	Test MAE	Test R^2	No. S	No. I
LR	SBM	6.204	-1.586	5.290	0.674	1263	1320
RR	SBM	5.268	0.689	5.176	0.697	1263	1320
Lasso	SBM	5.354	0.680	5.324	0.685	1263	1320
EN	SBM	5.269	0.689	5.176	0.697	1263	1320
RF	SBM	6.092	0.585	6.359	0.560	1263	1320
SVR	SBM	5.216	0.691	5.503	0.682	1263	1320
RVR	SBM	5.434	0.667	5.334	0.687	1263	1320
GPR	SBM	5.008	0.715	5.259	0.697	1263	1320
LR	VBM	4.839	0.726	5.034	0.699	1246	1794
RR	VBM	4.558	0.756	4.907	0.717	1246	1794
Lasso	VBM	4.539	0.762	4.805	0.724	1246	1794
EN	VBM	4.493	0.765	4.859	0.719	1246	1794
RF	VBM	5.383	0.663	5.277	0.641	1246	1794
SVR	VBM	4.361	0.776	4.368	0.764	1246	1794
RVR	VBM	4.562	0.755	4.721	0.723	1246	1794
GPR	VBM	4.278	0.781	4.317	0.766	1246	1794
LR	SM	8.427	0.154	8.478	0.146	1264	1815
RR	SM	4.898	0.722	4.937	0.728	1264	1815
Lasso	SM	5.122	0.697	5.306	0.688	1264	1815
EN	SM	5.045	0.703	5.206	0.701	1264	1815
RF	SM	7.792	0.288	7.332	0.364	1264	1815
SVR	SM	5.040	0.706	5.039	0.717	1264	1815
RVR	SM	5.034	0.704	5.077	0.708	1264	1815
GPR	SM	5.864	0.606	5.489	0.650	1264	1815

Supplementary Table 2: Pearson's r correlation between PAD and performance on neuropsychological tests. Negative Fluid Intelligence, positive Numeric Memory, positive Pairs Matching, negative prospective memory, positive Reaction Time, negative DSST, and positive TMT indicate worse performance. Abbreviations: confidence interval (CI), digit substitution test (DSST), trail making test (TMT).

Neuropsychological Test	PAD Correlation	95% CI	P Value	No. Subjects
Fluid Intelligence	-0.015	(-0.033, 0.004)	1.2e-01	11655
Numeric Memory	0.005	(-0.018, 0.028)	6.4e-01	7366
Pairs Matching	0.009	(-0.008, 0.026)	3.1e-01	12378
Prospective Memory	-0.023	(-0.041, -0.004)	1.7e-02	11001
Reaction Time	0.030	(0.012, 0.047)	7.9e-04	12387
DSST	-0.080	(-0.104, -0.054)	4.3e-11	6849
TMT A	0.053	(0.027, 0.078)	3.8e-05	6076
TMT B	0.076	(0.051, 0.103)	3.1e-09	6076
TMT B - A	0.050	(0.024, 0.075)	1.3e-04	5918

Subjects from the Icelandic sample diagnosed with neurodevelopmental or mental disorders were left out while training the brain age prediction method. This includes subjects with autism, bipolar disorder, schizophrenia, and intellectual disability. We now look for deviation in PAD in these subjects compared to the PAD of the healthy Icelanders.

A two sample t-test was used to test for the difference between the mean PAD of the neurodevelopmental and mental disorder cases and healthy controls. Before performing the analysis we first compute the PAD. In cases where subjects had multiple images the average PAD was used instead. In this particular instance the PAD was adjusted for age, gender, and total intracranial volume using a generalized additive model. Five correlation tests were performed, so we used a Bonferroni adjusted significance level $\alpha_{B1} = 0.05/5 \approx 0.01$. The controls are taken from the Icelandic test set (N=291) and their average PAD is 0.1. Most of the subjects in the schizophrenia group are young males. Therefore, in an effort to compare similar groups we performed an additional test where only the PAD of males under 35 years is compared. After applying this constraint there are 22 control subjects left and their average PAD is -0.6.

Supplementary Table 3: The average PAD difference between neurodevelopmental/mental disorder cases and controls from the test set estimated using a two sample t-test. Abbreviations: confidence interval (CI).

Cases	PAD Difference	95% CI	*P* Value	No. Cases	No. Controls
Schizophrenia	2.2	(1.2, 3.2)	3.5e-05	68	291
Schizophrenia (Male Under 35)	3.2	(1.4, 5.1)	1.1e-03	48	22
Intellectual Disability	1.5	(-0.5, 3.6)	1.2e-01	6	291
Autism	2.3	(-0.9, 5.6)	1.4e-01	10	291
Bipolar Disorder	0.4	(-0.6, 1.5)	4.0e-01	31	291

Supplementary Table 3 shows that PAD is higher in individuals with schizophrenia than in controls. This is consistent with findings from other studies [2, 3, 4, 5] that have looked at brain ageing of schizophrenia patients. Brain structure irregularities in schizophrenia, such as cortical thinning [6] and cerebral ventricular enlargement [7] also seen in healthy ageing [8, 9] may be driving the prediction. Why there are structural differences in brains from schizophrenia patients is not fully understood. Psychotic episodes and longtime use of antipsychotic drugs [10] probably contribute. Sequence variants conferring high-risk of the disease have also been shown to affect brain structure in controls [11].

Supplementary Table 4: SBM and VBM phenotypes associated rs1452628. Only structural brain phenotypes that reach genome-wide significance in the discovery set (UK Biobank) are listed (estimated using BOLT-LMM). In addition, the Pearson's r correlations between PAD and the SBM/VBM phenotypes are shown. Abbreviations: cerebrospinal fluid (CSF), gray matter (GM), gyrus (Gy), inferior (Inf), left hemisphere (LH), lobe (Lo), right hemisphere (RH), superior (Sup), thickness (Thk), volume (Vol).

	**rs1452628 Association**		**PAD Association**	
Phenotype	*P* Value	β	*P* Value	Pearson's r (%)
RH Sup parietal Gy CSF Vol	2.5e-33	-0.16	0.0	43.9
LH Sup parietal Gy CSF Vol	3.5e-31	-0.15	0.0	41.9
RH postcentral Gy CSF Vol	1.3e-28	-0.15	0.0	44.0
LH postcentral Gy CSF Vol	9.0e-26	-0.14	0.0	47.8
LH posterior cingulate Gy CSF Vol	2.5e-24	-0.13	0.0	44.4
RH posterior cingulate Gy CSF Vol	3.9e-24	-0.13	0.0	45.0
RH precentral Gy CSF Vol	4.5e-24	-0.13	0.0	41.8
LH precentral Gy CSF Vol	2.1e-21	-0.12	0.0	43.8
RH Inf lateral parietal Lo CSF Vol	5.0e-20	-0.12	0.0	45.0
LH middle frontal Gy CSF Vol	2.3e-19	-0.12	0.0	40.3
RH middle frontal Gy CSF Vol	2.6e-19	-0.12	0.0	37.5
LH Inf lateral parietal Lo CSF Vol	8.9e-18	-0.11	0.0	47.1
RH Sup frontal Gy CSF Vol	1.0e-16	-0.11	0.0	36.7
LH lateral occipital Lo CSF Vol	6.2e-16	-0.11	0.0	42.4
LH cuneus CSF Vol	3.3e-15	-0.10	0.0	42.8
LH Sup frontal Gy CSF Vol	4.1e-15	-0.10	0.0	39.6
LH precuneus Thk	2.3e-13	0.10	2.48e-109	-19.8
LH Sup parietal Thk	3.2e-13	0.10	1.43e-68	-15.7
RH Inf frontal Gy CSF Vol	3.6e-13	-0.10	0.0	42.3
RH cuneus CSF Vol	4.1e-13	-0.10	0.0	41.4
RH lateral occipital Lo CSF Vol	4.7e-13	-0.10	0.0	39.4
RH Sup parietal Thk	6.4e-12	0.09	2.2e-110	-19.9
LH anterior cingulate Gy CSF Vol	1.1e-11	-0.09	0.0	44.5
RH anterior cingulate Gy CSF Vol	8.1e-11	-0.09	0.0	43.3
RH Sup temporal Gy CSF Vol	1.6e-10	-0.08	0.0	48.9
RH precuneus Thk	6.4e-10	0.08	2.5e-109	-21.2
RH Sup occipital Gy GM Vol	2.2e-09	0.08	2.0e-142	-22.5

Supplementary Table 5: SBM and VBM phenotypes associated with rs2435204. Only structural brain phenotypes that reach genome-wide significance in the discovery set (UK Biobank) are listed (estimated using BOLT-LMM). In addition, the Pearson's r correlations between PAD and the SBM/VBM phenotypes are shown. Abbreviations: left hemisphere (LH), right hemisphere (RH), thickness (Thk), volume (Vol), white matter (WM).

	**rs2435204 Association**		**PAD Association**	
Phenotype	*P* Value	β	*P* Value	Pearson's r (%)
Total RH WM surface area	5.1e-17	-0.13	6.4e-27	-9.67
Total LH WM surface area	4.5e-16	-0.12	2.3e-30	-10.3
RH fusiform area	1.8e-15	-0.12	1.5e-22	-8.80
LH fusiform area	3.1e-14	-0.11	1.4e-20	-8.38
RH lateral occipital area	8.9e-12	-0.10	2.1e-7	-4.68
LH postcentral area	2.2e-10	-0.10	5.0e-2	-1.77
RH rostral middle frontal Thk	2.5e-10	0.10	3.3e-52	-13.7
LH lateral occipital area	9.8e-10	-0.09	5.6e-8	-4.90
RH lingual area	1.5e-09	-0.09	8.7e-5	-3.54
Total LH cerebral WM Vol	1.8e-09	-0.09	5.0e-98	-18.8

Supplementary Table 6: SBM and VBM phenotypes associated with rs2790099. Only structural brain phenotypes that reach genome-wide significance in the discovery set (UK Biobank) are listed (estimated using BOLT-LMM). In addition, the correlations between PAD and the SBM/VBM phenotypes are shown. Abbreviations: left hemisphere (LH), right hemisphere (RH), volume (Vol), white matter (WM).

	**rs2790099 Association**		**PAD Association**	
Phenotype	*P* Value	β	*P* Value	Pearson's r (%)
RH putamen WM Vol	4.0e-14	0.10	3.9e-61	-14.74
RH pallidum WM Vol	4.0e-12	0.09	1.6e-103	-19.23
LH putamen WM Vol	6.4e-12	0.09	4.3e-51	-13.45
RH thalamus WM Vol	1.2e-10	0.09	1.6e-44	-12.53
LH pallidum WM Vol	2.2e-10	0.09	1.5e-118	-20.57
LH thalamus WM Vol	5.0e-10	0.08	1.6e-36	-11.30
RH insula WM Vol	6.7e-09	0.08	4.2e-132	-21.71

Supplementary Table 7: SBM and VBM phenotypes associated with rs6437412. Only structural brain phenotypes that reach genome-wide significance in the discovery set (UK Biobank) are listed (estimated using BOLT-LMM). In addition, the Pearson's r correlations between PAD and the SBM/VBM phenotypes are shown. Abbreviations: cerebrospinal fluid (CSF), left hemisphere (LH), right hemisphere (RH), volume (Vol).

	**rs6437412 Association**		**PAD Association**	
Phenotype	*P* Value	β	*P* Value	Pearson's r (%)
LH middle frontal Gy CSF Vol	4.1e-14	-0.11	0.0	40.3
LH inferior lateral parietal lobe CSF Vol	1.1e-10	-0.09	0.0	47.8
RH middle frontal Gy CSF Vol	3.0e-10	-0.09	0.0	37.5
RH inferior lateral parietal lobe CSF Vol	4.5e-10	-0.09	0.0	45.0
RH precentral Gy CSF Vol	2.7e-09	-0.08	0.0	41.8
LH postcentral Gy CSF Vol	4.2e-09	-0.08	0.0	47.8

Supplementary Table 8: SBM and VBM phenotypes associated with rs2184968. Only structural brain phenotypes that reach genome-wide significance in the discovery set (UK Biobank) are listed (estimated using BOLT-LMM). In addition, the Pearson's r correlations between PAD and the SBM/VBM phenotypes are shown. Abbreviations: amygdaloid body (AmB), cerebrospinal fluid (CSF), gyrus (Gy), left hemisphere (LH), parahippocampal (ParHip), right hemisphere (RH), volume (Vol).

	**rs2184968 Association**		**PAD Association**	
Phenotype	*P* Value	β	*P* Value	Pearson's r (%)
RH cerebellum CSF Vol	4.3e-20	0.12	0.0	39.7
LH cerebellum CSF Vol	1.4e-19	0.12	0.0	39.2
RH brain stem CSF Vol	3.1e-16	0.10	0.0	37.0
LH brain stem CSF Vol	3.9e-13	0.10	0.0	37.0
LH AmB and ParHip Gy CSF Vol	1.1e-09	0.08	3.6e-316	33.2
4th Ventricle Vol.	4.6e-09	0.08	1.4e-69	15.8

Supplementary Notes

Supplementary Note 1

Fluid intelligence: Participants are asked to solve problems that require logic and reasoning ability, independent of acquired knowledge and have 2 minutes to complete as many questions as possible. More information can be found here: http://biobank.ctsu.ox.ac.uk/crystal/label.cgi?id=100027

Supplementary Note 2

Numeric memory: Participants are shown a 2-digit number which then disappears and after certain period are asked to recall the number. The test starts with a 2-digit number and becomes 1-digit longer each time they remember correctly up to a maximum of 12 digits. More information can be found here: http://biobank.ctsu.ox.ac.uk/crystal/label.cgi?id=100029

Supplementary Note 3

Visual memory: Participants are asked to memorize the position of as many matching pairs of cards as possible. The cards are then turned face down and the participant is asked to find as many pairs as possible. More information can be found here: http://biobank.ctsu.ox.ac.uk/crystal/label.cgi?id=100030

Supplementary Note 4

Prospective memory: Participants are shown four colored shapes and asked to touch a square. They should remember that earlier they were asked to touch the orange circle instead. More information can be found here: http://biobank.ctsu.ox.ac.uk/crystal/label.cgi?id=100031

Supplementary Note 5

Simple processing speed: Participants play 12 rounds of the card-game 'Snap' to assess reaction time. They are shown two cards at a time; if both cards are the same, they press a button as quickly as possible. More information can be found here: http://biobank.ctsu.ox.ac.uk/crystal/label.cgi?id=100032

Supplementary Note 6

Complex processing speed: Participants are asked to solve a digit symbol substitution test (DSST). They are presented with a series of grids in which symbols are to be matched to numbers according to a key presented on the screen. More information can be found here: http://biobank.ctsu.ox.ac.uk/crystal/label.cgi?id=122

Supplementary Note 7

Visual attention: Participants are asked to solve a trail making test (TMT) of type A and B. They are presented with a series of labeled circles and instructed to touch them according to a particular ordering rule. More information can be found here: https://biobank.ctsu.ox.ac.uk/crystal/label.cgi?id=121

Supplementary References

[1] H. Stefansson, A. Helgason, G. Thorleifsson, V. Steinthorsdottir, G. Masson, J. Barnard, A. Baker, A. Jonasdottir, A. Ingason, V.G. Gudnadottir, N. Desnica, A. Hicks, A. Gylfason, D.F. Gudbjartsson, G.M. Jonsdottir, J. Sainz, K. Agnarsson, B. Birgisdottir, S. Ghosh, A. Olafsdottir, J.B. Cazier, K. Kristjansson, M.L. Frigge, J.R. Gulcher T.E. Thorgeirsson, A. Kong, and K. Stefansson. A common inversion under selection in europeans. *Nature Genetics*, 37:129–137, (2005).

[2] Igor Nenadić, Maren Dietzek, Kerstin Langbein, Heinrich Sauer, and Christian Gaser. Brainage score indicates accelerated brain aging in schizophrenia, but not bipolar disorder. *Psychiatry Research: Neuroimaging*, 266:86–89, (2017).

[3] Hugo G Schnack, Neeltje EM Van Haren, Mireille Nieuwenhuis, Hilleke E Hulshoff Pol, Wiepke Cahn, and René S Kahn. Accelerated brain aging in schizophrenia: a longitudinal pattern recognition study. *American Journal of Psychiatry*, 173(6):607–616, (2016).

[4] Nikolaos Koutsouleris, Christos Davatzikos, Stefan Borgwardt, Christian Gaser, Ronald Bottlender, Thomas Frodl, Peter Falkai, Anita Riecher-Rössler, Hans-Jürgen Möller, Maximilian Reiser, et al. Accelerated brain aging in schizophrenia and beyond: a neuroanatomical marker of psychiatric disorders. *Schizophrenia bulletin*, 40(5):1140–1153, (2013).

[5] Tobias Kaufmann, Dennis van der Meer, Nhat Trung Doan, Emanuel Schwarz, Martina J Lund, Ingrid Agartz, Dag Alnæs, Deanna M Barch, Ramona Baur-Streubel, Alessandro Bertolino, et al. Common brain disorders are associated with heritable patterns of apparent aging of the brain. *Nature neuroscience*, 22(10):1617–1623, (2019).

[6] Neeltje EM van Haren, Hugo G Schnack, Wiepke Cahn, Martijn P van den Heuvel, Claude Lepage, Louis Collins, Alan C Evans, Hilleke E Hulshoff Pol, and René S Kahn. Changes in cortical thickness during the course of illness in schizophrenia. *Archives of general psychiatry*, 68(9):871–880, (2011).

[7] Daniel R Weinberger, E Fuller Torrey, Andreas N Neophytides, and Richard Jed Wyatt. Lateral cerebral ventricular enlargement in chronic schizophrenia. *Archives of General Psychiatry*, 36(7): 735–739, (1979).

[8] David H Salat, Randy L Buckner, Abraham Z Snyder, Douglas N Greve, Rahul SR Desikan, Evelina Busa, John C Morris, Anders M Dale, and Bruce Fischl. Thinning of the cerebral cortex in aging. *Cerebral cortex*, 14(7):721–730, (2004).

[9] Jeffrey A Kaye, Charles DeCarli, Jay S Luxenberg, and Stanley I Rapoport. The significance of age-related enlargement of the cerebral ventricles in healthy men and women measured by quantitative computed x-ray tomography. *Journal of the American Geriatrics Society*, 40(3):225–231, (1992).

[10] J Moncrieff and J Leo. A systematic review of the effects of antipsychotic drugs on brain volume. *Psychological medicine*, 40(9):1409–1422, (2010).

[11] H. Stefansson and et al. CNVs conferring risk of autism or schizophrenia affect cognition in controls. *Nature*, 505:361–366, (2014).
