# Supplementary material for: Brain age prediction using deep learning uncovers associated sequence variants
Source: Nat Commun. 2019 Nov 27;10:5409. doi: 10.1038/s41467-019-13163-9 (PMC6881321; doi:10.1038/s41467-019-13163-9)
Supplement: Supplementary file 2 — Description of Additional Supplementary Files [file 41467_2019_13163_MOESM2_ESM.pdf]

Description of Additional Supplementary Files

File Name: Supplementary Data 1

Description: Tables containing the names of SBM and VBM variables used for brain age prediction.
