# Supplementary material for: Brain age prediction using deep learning uncovers associated sequence variants
Source: Nat Commun. 2019 Nov 27;10:5409. doi: 10.1038/s41467-019-13163-9 (PMC6881321; doi:10.1038/s41467-019-13163-9)
Supplement: Supplementary file 5 — Reporting Summary [file 41467_2019_13163_MOESM5_ESM.pdf]

nature research

Corresponding author(s): M.O. Ulfarsson
K. Stefansson

Last updated by author(s): Oct 16, 2019

Reporting Summary

Nature Research wishes to improve the reproducibility of the work that we publish. This form provides structure for consistency and transparency in reporting. For further information on Nature Research policies, see Authors & Referees and the Editorial Policy Checklist.

Statistics

For all statistical analyses, confirm that the following items are present in the figure legend, table legend, main text, or Methods section.

n/a	Confirmed	
☐	☒	The exact sample size (n) for each experimental group/condition, given as a discrete number and unit of measurement
☐	☒	A statement on whether measurements were taken from distinct samples or whether the same sample was measured repeatedly
☐	☒	The statistical test(s) used AND whether they are one- or two-sided<br>*Only common tests should be described solely by name; describe more complex techniques in the Methods section.*
☐	☒	A description of all covariates tested
☐	☒	A description of any assumptions or corrections, such as tests of normality and adjustment for multiple comparisons
☐	☒	A full description of the statistical parameters including central tendency (e.g. means) or other basic estimates (e.g. regression coefficient) AND variation (e.g. standard deviation) or associated estimates of uncertainty (e.g. confidence intervals)
☐	☒	For null hypothesis testing, the test statistic (e.g. F, t, r) with confidence intervals, effect sizes, degrees of freedom and P value noted<br>*Give P values as exact values whenever suitable.*
☒	☐	For Bayesian analysis, information on the choice of priors and Markov chain Monte Carlo settings
☒	☐	For hierarchical and complex designs, identification of the appropriate level for tests and full reporting of outcomes
☐	☒	Estimates of effect sizes (e.g. Cohen's d, Pearson's r), indicating how they were calculated

Our web collection on statistics for biologists contains articles on many of the points above.

Software and code

Policy information about availability of computer code

Data collection	No software was used to collect data.
Data analysis	In-house deCODE genetics software was used for genetic association analyses. The Computational anatomy toolbox (CAT12) and FreeSurfer (V6.0.0) was used to preprocess all MRI images used in this study. Custom code was written to implement the brain age prediction method described in the manuscript. Additionally, custom code was used to perform statistical tests and prepare plots included in the manuscript.

For manuscripts utilizing custom algorithms or software that are central to the research but not yet described in published literature, software must be made available to editors/reviewers. We strongly encourage code deposition in a community repository (e.g. GitHub). See the Nature Research guidelines for submitting code & software for further information.

Data

Policy information about availability of data

All manuscripts must include a data availability statement. This statement should provide the following information, where applicable:

- Accession codes, unique identifiers, or web links for publicly available datasets
- A list of figures that have associated raw data
- A description of any restrictions on data availability

The data used in the analyses for this publication are not publicly available due to information, contained within them, that could compromise research participant privacy. The authors declare that the data supporting the findings of this study are available within the article, its supplementary information, and upon request.

The IXI MRI dataset is freely available (https://brain-development.org/ixi-dataset/).

Researchers can apply to use the UK Biobank resource for health related research that is in the public interest. A guide to applying for access to the UK Biobank is provided here: https://www.ukbiobank.ac.uk/register-apply/

Field-specific reporting

Please select the one below that is the best fit for your research. If you are not sure, read the appropriate sections before making your selection.

☒ Life sciences ☐ Behavioural & social sciences ☐ Ecological, evolutionary & environmental sciences

For a reference copy of the document with all sections, see nature.com/documents/nr-reporting-summary-flat.pdf

Life sciences study design

All studies must disclose on these points even when the disclosure is negative.

Sample size	All data available to us in the Icelandic, IXI, and UK Biobank datasets were used. No sample size calculations were performed beforehand. The sample size was determined to be sufficient based on results from statistical significance tests.
Data exclusions	During training of the brain age prediction method, subjects diagnosed with neurological or neurodevelopmental disorders such as autism, bipolar disorder, intellectual disability, or schizophrenia and subjects with any copy number variations previously associated with neurodevelopmental or psychiatric disorder were excluded. This was done in an effort to keep the training set as healthy as possible in order to prevent introducing any pathalogical brains during training that could skew the models estimates.<br><br>Subjects of non British ancestry were excluded from the analysis of the UK Biobank to prevent introducing any unexpected effects caused by differences between natives and foreigners.
Replication	We took steps to test to test the GWAS results in a replication set of 4453 subjects. The five reported sequence variants also associate with PAD in this replication set.
Randomization	The samples were grouped according sequence variant carrier status.
Blinding	Both the psychologists performing the cognitive assessment and the MRI recruitment staff were blind to genotype status.

Reporting for specific materials, systems and methods

We require information from authors about some types of materials, experimental systems and methods used in many studies. Here, indicate whether each material, system or method listed is relevant to your study. If you are not sure if a list item applies to your research, read the appropriate section before selecting a response.

Materials & experimental systems

n/a	Involved in the study	
☒	☐	Antibodies
☒	☐	Eukaryotic cell lines
☒	☐	Palaeontology
☒	☐	Animals and other organisms
☐	☒	Human research participants
☒	☐	Clinical data

Methods

n/a	Involved in the study	
☒	☐	ChIP-seq
☒	☐	Flow cytometry
☐	☒	MRI-based neuroimaging

Human research participants

Policy information about studies involving human research participants

Population characteristics	Participants in this study are all of Icelandic or British nationality.
Recruitment	The Icelandic participants in this study were recruited by deCODE genetics for a schizophrenia study. The UK Biobank oversaw the recruitment of subjects of British nationality.
Ethics oversight	Approval for the aforementioned schizophrenia study was obtained from the National Bioethics Committee of Iceland and the Icelandic Data Protection Authority. Written informed consent was obtained from all participants or their guardians before blood samples or phenotypic data were obtained. All sample identifiers were encrypted in accordance with the regulations of the Icelandic Data Protection Authority.<br><br>Information about ethics oversight in the UK Biobank can be found here: https://www.ukbiobank.ac.uk/ethics/

Note that full information on the approval of the study protocol must also be provided in the manuscript.

Magnetic resonance imaging

Experimental design

Design type	Structural MRI imaging (sMRI)
Design specifications	Not applicable, fMRI was not a part of this study.
Behavioral performance measures	Not applicable, fMRI was not a part of this study.

Acquisition

Imaging type(s)	T1 weighted structural MRI
Field strength	Icelandic data:<br>1.5T (Phillips Achieva)<br>1.5T (Siemens Magnetom Aera)<br><br>IXI data:<br>3T (Hammersmith Hospital)<br>1.5T (Guy's Hospital)<br>1.5T (Institute of Psychiatry)<br><br>UK Biobank:<br>3T (Siemens Skyra)
Sequence & imaging parameters	The Icelandic MRIs were imaged using a sagittal 3D fast T1-weighted gradient echo sequence. The imaging parameters of the two scanners are listed below.<br><br>Philips Achieva : repetition time (TR) = 8.6 ms, echo time (TE) = 4.0 ms, flip angle = 8°, slice thickness = 1.2 mm, matrix = 192 × 192, field of view (FOV) = 240 × 240 mm, orientation = sagittal<br><br>Siemens Magnetom Aera: repetition time (TR) = 2400 ms, echo time (TE) = 3.54 ms, flip angle = 8 °, slice thickness = 1.2 mm, matrix = 192 × 192, field of view = 240 × 240 mm, orientation = sagittal<br><br>Information about the IXI imaging parameters can be found here: https://brain-development.org/ixi-dataset/<br><br>Details about the UK Biobank MRI image parameters is available here: https://biobank.ctsu.ox.ac.uk/crystal/docs/brain_mri.pdf
Area of acquisition	Whole brain scans were used.

Diffusion MRI ☐ Used ☒ Not used

Preprocessing

Preprocessing software	The computational anatomy toolbox (CAT12) was used for skull-stripping, denoising, registration, and segmentation. More information about the preprocessing steps performed by CAT12 can be found on page 51 in its manual (www.neuro.uni-jena.de/cat12/CAT12-Manual.pdf).<br><br>Additionally, FreeSurfer (V6.0.0) was used for extraction of surface-based morphometry features (https://surfer.nmr.mgh.harvard.edu/fswiki).
Normalization	The T1 weighted MRIs were registered to MNI space with deformable registration using the DARTEL algorithm.
Normalization template	The data was normalized to an already existing DARTEL template derived from 555 healthy control subjects from the IXI dataset.
Noise and artifact removal	The MRIs were denoised using CAT12 (www.neuro.uni-jena.de/cat12/CAT12-Manual.pdf).
Volume censoring	Volumes were not censored.

Statistical modeling & inference

Model type and settings	A brain age prediction is calculated for each subject using a convolutional neural network, these predictions are then used to test for association (using regression and t-tests) with phenotypes and genotypes.
Effect(s) tested	Not applicable, fMRI was not a part of this study.

Specify type of analysis: ☒ Whole brain ☐ ROI-based ☐ Both

Statistic type for inference (See Eklund et al. 2016)	Not applicable, fMRI was not a part of this study.
Correction	Not applicable, fMRI was not a part of this study.

Models & analysis

n/a	Involved in the study
☒	☐ Functional and/or effective connectivity
☒	☐ Graph analysis
☐	☒ Multivariate modeling or predictive analysis

Multivariate modeling and predictive analysis	The brain age prediction model is a convolutional neural network that takes preprocessed T1 weighted MRIs (T1 weighted image, Jacobian map, gray matter segmented image, and white matter segmented image) as inputs and outputs an age prediction. The metric used to train and evaluated the model is the mean absolute error between predicted brain age and chronological age. The model performance is evaluated on images left out during training (test set).
